# Sambamba: fast processing of NGS alignment formats

**DOI:** 10.1093/bioinformatics/btv098

**Published:** 2015-02-19

**Authors:** Artem Tarasov, Albert J. Vilella, Edwin Cuppen, Isaac J. Nijman, Pjotr Prins

**Affiliations:** ^1^Department of Statistical Simulation, St. Petersburg State University, St. Petersburg, Russia, ^2^Illumina Cambridge, Cambridge, UK, ^3^Hubrecht Institute, Royal Netherlands Academy of Arts and Sciences, Utrecht, The Netherlands, ^4^Department of Medical Genetics, Institute for Molecular Medicine, University Medical Centre Utrecht, Utrecht, The Netherlands and ^5^Department of Nematology, Wageningen University, Wageningen, The Netherlands

## Abstract

**Summary:** Sambamba is a high-performance robust tool and library for working with SAM, BAM and CRAM sequence alignment files; the most common file formats for aligned next generation sequencing data. Sambamba is a faster alternative to samtools that exploits multi-core processing and dramatically reduces processing time. Sambamba is being adopted at sequencing centers, not only because of its speed, but also because of additional functionality, including coverage analysis and powerful filtering capability.

**Availability and implementation:** Sambamba is free and open source software, available under a GPLv2 license. Sambamba can be downloaded and installed from http://www.open-bio.org/wiki/Sambamba.

Sambamba v0.5.0 was released with doi:10.5281/zenodo.13200.

**Contact**: j.c.p.prins@umcutrecht.nl

## 1. Introduction

Processing speed matters, not only for diagnostics, but also for analysis and sharing of computational resources. Next-generation sequencing (NGS) is increasingly used as a genetic screening tool in diagnostics (Gullapalli *et* *al.*, 2012) and reducing time from sample intake to test result/diagnosis potentially saves lives. Introducing multi-core processing can accelerate steps in a pipeline when the CPU is the bottleneck (Trelles *et* *al.*, 2011).

**Fig. 1.**
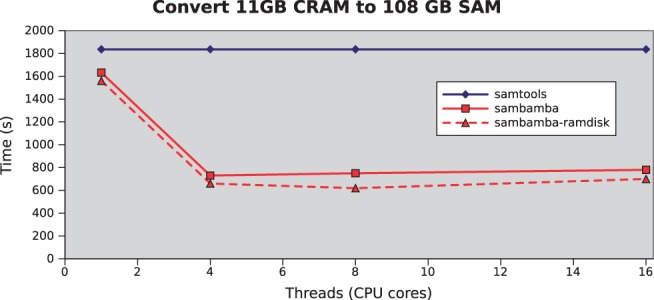
Processing speed comparison of samtools and sambamba. Wall-clock time (s) versus number of threads to convert an 11-GB CRAM (1000 genomes HG00110) to 108-GB SAM. With Samtools, view is bound to a single thread at CPU 90%. With Sambamba, IO gets saturated at approximately CPU 250%. When using a faster RAM-disk, IO gets saturated at approximately CPU 350%. For samtools a RAM-disk makes no difference. When adding more threads, performance reproducibly degrades because of CPU cache contention. All timings were performed on a server-class machine with 512 GB of RAM and 48 CPU cores (4 × 12-core AMD Opteron(tm) Processor 6174 @2.2 Ghz with 6 Mb L2 cache) Samtools version v1.0-15 using htslib v1.0-1 and sambamba v0.5.0 compiled with the LLVM D-compiler v0.14.0.

Since its introduction by the 1000 Genomes Project (Siva, 2008), the sequence alignment/map format (SAM) and its compressed binary counterpart (BAM) have become the *de facto* file formats used for storing and distributing NGS data. Samtools is the original tool for SAM/BAM files processing, including data extraction and filtering (Li *et* *al.*, 2009). Recently, samtools added the CRAM format as a compressed alternative to SAM/BAM (Cochrane *et* *al.*, 2013). While samtools exploits the speed of the low-level C programming language and uses streamed data for efficiency, it has limited support for parallel processing (Fig. 1). Samtools has inspired a number of other BAM processors, notably Picard (Picard, 2009), samblaster (Faust and Hall, 2014), biobambam (Tischler and Leonard, 2014) and Scramble (Bonfield, 2014), each of which is either slower than samtools, or offers a subset of its functionality.

To accelerate analysis pipelines we created Sambamba, a new incarnation of samtools that fully utilizes parallel processing. Sambamba (which means ‘parallel’ in Swahili) is written in the D programming language, a modern programming language with run-time performance similar to that of C (Alexandrescu, 2010). D has powerful abstractions for parallel computing which make it possible to scale computations with the number of cores (Fig. 1). When running a Human cancer exome SNV calling pipeline on the results of a single Illumina HiSeq 2500 flowcell in fast mode (2000 genes, 300 million reads, 100 bp read length and average read depth of 100 for six samples) following standard best practice guidelines, the bioinformatics processing time was reduced from 2 h to 30 min by replacing Picard markdup and samtools index, flagstat, merge and view.

## 2. Features

Sambamba introduces full parallelized data processing of SAM, BAM and CRAM files. Sambamba primarily uses D’s parallel processing capabilities. For CRAM support the htslib C-library was linked against (Bonfield, 2014). And for mpileup support the original samtools program is called in map-reduce fashion. This resulted in improved processing speed on multi-core computers (Table 1). Sambamba is most effective on machines where CPU utilization is the constraining factor (Fig. 1). The gain may therefore be limited on cluster setups where shared storage is a bottleneck (e.g. Trelles *et* *al*., 2011).

**Table 1.** Examples of processing of 31-GB BAM and matching 11-GB CRAM of HG00110 with sambamba and samtools. Wall-clock time (t in seconds) reflects improved analysis time. CPU (×100%) reflects effective multi-core utilisation. See Figure 1 caption for description of hardware, software and measurements

	samtools t(s) CPU%		Sambamba t(s) CPU%		Speedup
BAM view	1506		429	785	3.5×
Filter^a^	195		35	471	3.5×
Sort	12 288	396%	1265	945	10×
Index	577		137	562	4×
Markdup^b^	5220		2296	278	2×
Merge	3090	571%	2247	1015	1.5×
mpileup^c^	7750		584	4409	13×
BAM to CRAM	4354		640	796	7×
CRAM to SAM	1850		729	347	2.5×
CRAM index	9		9		=

^a^Filter on *q* > 30 and *Chr*1.

^b^For markdup samtools v0.19 was used.

^c^mpileup to VCF on 2GB BAM of *Chr*1 only.

Compatibility: Sambamba is a robust replacement for the commonly used samtools commands: index, sort, view, mpileup, markdup, merge and flagstat. The output of sambamba compares to that of samtools, except for markdup, where the Picard ‘sum of base qualities’ method was chosen. Sambamba’s RAM utilization compares to that of samtools; only with sort sambamba uses significantly less RAM.

New functionality: Sambamba adds new functionality compared with existing tools. To be able to calculate coverage statistics, read depth analysis was added. To speed up splitting BAM files, slice was added which copies large regions without decompression. And when a BED file is supplied to view, the index is used to decompress only those regions that are actually visited.

To further shorten processing time, index files are created on the fly by sort, view, markdup and merge. And to combine multiple steps into one, powerful filtering with logic operators and regular expressions was added. For example, to filter on mapping quality and CIGAR

mapping_quality >= 30 and cigar = ∼ / ∧ \d+M1I\d+M$/

Finally, to make it easier to process results, sambamba view can generate output in the standard JSON format.

Source code: Sambamba abides by the rules of the ‘Small tools MANIFESTO for Bioinformatics’ (Prins *et* *al.*, 2014). The sambamba source code is extensible and maintainable. For SAM parsing we opted for Ragel, a finite-state machine compiler, which generates a fast look-ahead parser with input validation, making the code base even more compact (Thurston, 2006). Sambamba uses a unit testing framework with continuous integration testing, so that existing functionality is validated every time the code base is changed.

## 3. Conclusion

Sambamba is a software engineering example that shows how to make effective use of the D programming language and multi-core computers to reduce the time needed to get from sample to result. Whole genome sequencing and growing sample numbers make such performance improvements increasingly relevant.
